# Donor–Acceptor Co-Adsorption Ratio Controls the Structure and Electronic Properties of Two-Dimensional Alkali–Organic Networks on Ag(100)

**DOI:** 10.1021/acs.jpcc.2c08688

**Published:** 2023-01-26

**Authors:** B. Sohail, P. J. Blowey, L. A. Rochford, P. T. P. Ryan, D. A. Duncan, T.-L. Lee, P. Starrs, G. Costantini, D. P. Woodruff, R. J. Maurer

**Affiliations:** † Department of Chemistry, University of Warwick, CoventryCV4 7AL, U.K.; ‡ Department of Physics, University of Warwick, CoventryCV4 7AL, U.K.; § Diamond Light Source, Harwell Science and Innovation Campus, DidcotOX11 0DE, U.K.; ∥ School of Chemistry, University of Birmingham, BirminghamB15 2TT, U.K.; ⊥ Department of Materials, Imperial College, London, LondonSW7 2AZ, U.K.; # School of Chemistry, University of St. Andrews, St. AndrewsKY16 9AJ, U.K.

## Abstract

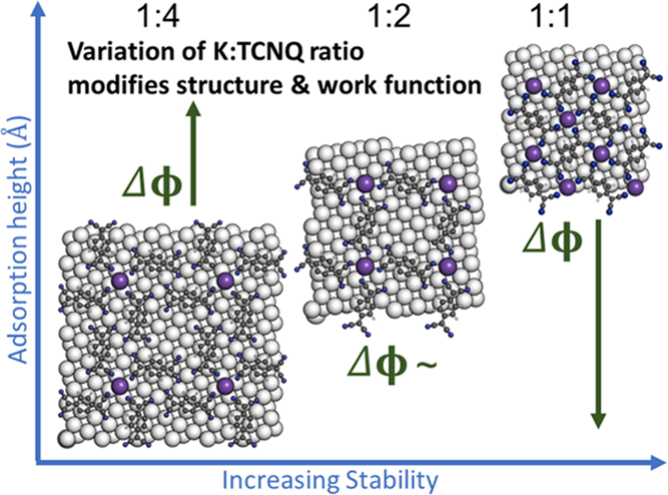

The results are presented of a detailed combined experimental and theoretical investigation of the influence of coadsorbed electron-donating alkali atoms and the prototypical electron acceptor molecule 7,7,8,8-tetracyanoquinodimethane (TCNQ) on the Ag(100) surface. Several coadsorption phases were characterized by scanning tunneling microscopy, low-energy electron diffraction, and soft X-ray photoelectron spectroscopy. Quantitative structural data were obtained using normal-incidence X-ray standing wave (NIXSW) measurements and compared with the results of density functional theory (DFT) calculations using several different methods of dispersion correction. Generally, good agreement between theory and experiment was achieved for the quantitative structures, albeit with the prediction of the alkali atom heights being challenging for some methods. The adsorption structures depend sensitively on the interplay of molecule–metal charge transfer and long-range dispersion forces, which are controlled by the composition ratio between alkali atoms and TCNQ. The large difference in atomic size between K and Cs has negligible effects on stability, whereas increasing the ratio of K/TCNQ from 1:4 to 1:1 leads to a weakening of molecule–metal interaction strength in favor of stronger ionic bonds within the two-dimensional alkali–organic network. A strong dependence of the work function on the alkali donor–TCNQ acceptor coadsorption ratio is predicted.

## 1 Introduction

The use of molecular films as components for organic electronics has attracted growing interest in recent years.^1^ Thin films composed of organic molecules or polymers are already applied within commercially available technologies such as organic light-emitting diodes (OLED)^2,3^ and organic photovoltaics (OPVs).^4,5^ A significant challenge is posed by the interface formed between organic molecules and metal electrodes, and the electronic properties of the interface have the potential to significantly affect device functionality. Charge injection barriers can, in particular, have a deleterious effect on device performance, so a crucial aspect of organic semiconductor design is to optimize the energy-level alignment across the metal–organic interface. The energy-level alignment is strongly influenced by the presence of interfacial dipoles that are modulated by many contributions such as charge transfer,^6^ the “push back effect,”^7−10^ bonding, and intrinsic dipoles.^11^

A variety of model systems have been investigated to understand such effects at the atomic scale and the implications they have for the interface. These include, but are not limited to, the use of self-assembled monolayers (SAMs),^12−14^ doping organic semiconductors with strong acceptor species,^15−18^ or deposition of (sub)monolayers of strong acceptors (or donors) at metal surfaces.^19−24^ These systems, in particular the last group, have provided insight into adsorption-induced substrate work function changes. However, it is still considered a challenge to improve reliably the energy-level alignment across the interface.^1,25−27^ Thus, to move away from a trial-and-error approach to optimize the fabrication of thin films and toward a more rational design process, we must establish a deeper understanding of the interactions at the organic–inorganic interface. While state-of-the-art electronic structure calculations have the ability to predict structure, stability, and dipole formation at the interface,^28^ their ability to do so accurately and robustly for a variety of systems needs to be carefully validated.

A potential route to tuning energy-level alignment at the interface is the coupling of strong, organic acceptor molecules coadsorbed with alkali atoms to act as a spacer layer between the metal electrode and the organic semiconductor.^29−31^ Coadsorption with alkali atoms will affect not only the interfacial dipole (and consequently the level alignment) but also the adsorption structure when forming two-dimensional metal–organic frameworks (2D-MOF). Various phases of K atoms coadsorbed with perylenetetracarboxylic dianhydride (PTCDA) on Ag(111) have previously been studied.^32^ The incorporation of K atoms into the overlayer leads to profound structural arrangements which were identified through scanning tunneling microscopy (STM), ST[H]M, and density functional theory (DFT). Additionally, these phases were explored in a joint experimental and theoretical approach, which comprehensively detailed the change in electronic properties as a function of K coverage.^33^

Understanding the parameters that lead to the formation of such phases can aid the use of alkali doping techniques to control the surface properties of metal–organic interfaces. A particularly relevant molecule in this context is 7,7,8,8-tetracyanoquinodimethane (TCNQ), which is a prototypical electron acceptor that can be used as an additive to interfaces to lower the energy barrier for electron transfer from the metal into the semiconductor. TCNQ is also capable of forming highly conductive charge transfer salts in combination with suitable electron donor molecules.^34−38^ Moreover, TCNQ adsorbed on Ag surfaces has been shown to form a diverse range of phases on the (111)^39,40^ and (100)^41^ terminations, some of which have been found to incorporate Ag metal adatoms into the adsorbate network, inducing the formation of a two-dimensional metal–organic framework (2D-MOF). In particular, when co-deposited with alkali metals, TCNQ forms a number of different two-dimensional networks as a function of overall coverage and alkali/TCNQ ratio, representing an ideal system to study the controllable synthetic parameters for 2D-MOF formation. Floris et al.,^42^ using non-dispersion-corrected density functional theory (DFT), first drew attention to the possibility that alkali atom coadsorption with TCNQ on Ag(100) could be used to tune the interfacial dipole and hence the barrier to charge transfer at a metal–organic interface.

The inherent versatility of TCNQ is exemplified on Ag(111) as it was shown that the network composed of TCNQ and Ag adatoms formed by TCNQ adsorption,^39^ is disrupted by deposition of K atoms and subsequent annealing.^43^ This treatment results in the formation of a new phase with stoichiometry K_2_TCNQ, which we have shown previously^43^ to form a 2D organic salt with significant intralayer cohesion and, compared to an adlayer network of TCNQ and Ag adatoms, a weakened interaction with the substrate. In addition, the presence of K also led to a significant modification of the work function and the electronic structure. It is, in particular, these modifications of the electronic structure that state-of-the-art density functional theory calculations seek to quantify.^44^

Robust theoretical modeling is crucial to understand the stability and electronic properties of metal–organic interfaces. It is well established that long-range dispersion interactions must be accounted for to achieve accurate predictions of the structure and stability of such systems.^45−47^ In cases of strong charge transfer, such as in these electron donor/acceptor pairs, long-range dispersion needs to account for the change in polarizability that occurs during charge transfer. We have previously studied this in the context of Ag(111)-K_2_TCNQ and have found that the well-established surface-screened Tkatchenko–Scheffler vdW method (vdW^surf^) for DFT does not fully capture this effect. As a remedy to account for this charge transfer, we employed a rescaling scheme of the atomic polarizability and C_6_ coefficient.^43,48^ It has not yet been assessed if more recent beyond-pairwise dispersion correction methods, such as the nonlocal many-body dispersion method (MBD-NL),^49^ are able to fully capture the interplay of charge transfer effects and long-range dispersion found for donor–acceptor networks at metal surfaces; this we address as part of this manuscript.

Here, we present the results of a detailed combined experimental and theoretical investigation of four different experimentally realized TCNQ/alkali coadsorption phases on the Ag(100) surface. Two of these, with stoichiometry K(TCNQ)_4_ and Cs(TCNQ)_4_, are phases that were previously investigated in the purely computational study of Floris et al.,^42^ while the existence of the Cs(TCNQ)_4_ was previously identified by scanning tunneling microscopy (STM).^50^ Our experimental investigation includes not only the characterization of several different coadsorption phases on Ag(100) by STM, low-energy electron diffraction (LEED), and soft X-ray photoelectron spectroscopy (SXPS) but also quantitative structural information on the heights of the coadsorbed species above the surface using normal-incidence X-ray standing waves (NIXSW) measurements. The paper is structured as follows: we first describe the experimental fabrication and characterization of the different 2D-MOF phases on Ag(100), before developing structural models that achieve quantitative agreement between NIXSW measurements and DFT predictions. Having established a full atomistic understanding of the 2D-MOF structures, we compare trends in the different 2D-MOF phases in terms of structure, stability, and electronic properties upon incorporation of different alkali atoms (K vs Cs) and upon changing the coadsorption ratio between donor and acceptor (1:4, 1:2, 1:1). We find that the coadsorption ratio sensitively affects the work function and the balance of interactions within the 2D-MOF and between substrate and 2D-MOF. This enables us to extract some general principles for the fabrication of 2D-MOF layers on metallic surfaces.

## 2 Methods

### 2.1 Experimental Methods

Experimental characterization of the coadsorption phases of TCNQ with Cs and K (and also Na) on Ag(100) was performed using STM and low-current (microchannel plate) low-energy electron diffraction (MCP-LEED) at room temperature in a UHV surface science chamber at the University of Warwick, but also by MCP-LEED and soft X-ray photoelectron spectroscopy (SXPS) in the UHV end-station of beamline I09 of the Diamond Light Source^51^ used for the NIXSW measurements. Well-ordered clean Ag(100) samples were cleaned *in situ* by cycles of sputtering (Ar^+^, 1 keV) and annealing in both chambers. Single-molecular monolayer structures were prepared by vacuum deposition of TCNQ from molecular beam epitaxy sources installed in the chambers, with alkali deposition being effected from resistively heated SAES “getter” sources. STM images, recorded in constant current mode using electrochemically etched polycrystalline tungsten tips, were plane corrected and flattened using the open-source image-processing software Gwyddion.^52^ To form the alkali-TCNQ coadsorption phases, TCNQ was first deposited on a clean Ag(100) surface to form the commensurate  phase using the preparation method described previously.^41^ The alkali atoms were then deposited to increasing coverages onto the sample at room temperature. The highest K coverage ordered phase required annealing to ∼300 °C to produce a well-ordered surface (see Table S1).

For the NIXSW measurements (also at room temperature), the X-ray absorption probabilities of the C and N atoms of TCNQ, as well as at the coadsorbed K and Cs atoms, were monitored by recording the intensity of the C 1s, N 1s, K 2p, and Cs 3d photoelectron spectral peaks. These “hard” X-ray spectra, as well as the high-resolution soft X-ray spectra, were collected using a VG Scienta EW4000 HAXPES hemispherical electron analyzer mounted at 90° to the incident photon beam, while sweeping the photon energy through the (200) Bragg condition at near-normal incidence to the surface. Both the high-resolution SXP spectra (measured using “soft” X-rays at photon energies of ca. 400–900 eV) and the “HAXPE” spectra recorded in NIXSW experiments (photoemission spectra at the “harder” X-ray energies corresponding to the Bragg (200) scattering condition at ∼3040 eV) were fitted using the CasaXPS software package to allow chemical-state specific NIXSW data to be extracted. Fitting of the NIXSW absorption profiles to extract the structural parameters included taking account of the nondipolar effects on the angular dependence of the photoemission, using values for the backward-forward asymmetry parameter *Q*,^53^ obtained from theoretical angular distribution parameters.^54^

### 2.2 Computational Methods

The Fritz-Haber Institute *ab initio* molecular simulations package (FHI-aims)^55^ was employed to perform density functional theory calculations. We use the generalized gradient approximation (GGA) exchange-correlation functional by Perdew, Burke, and Ernzerhof (PBE)^56^ coupled with dispersion correction schemes to account for van der Waals contributions to the total energy. We employed the surface-screened Tkatchenko–Scheffler van der Waals scheme (PBE+vdW^surf^)^57^ with and without the use of a manual rescaling procedure of the free atom polarizability and C_6_ coefficient as reported by Blowey et al.^43^ We also employed the recently proposed nonlocal many-body dispersion scheme (PBE+MBD-NL),^58^ which requires no such rescaling.

The adsorption structures were modeled as a periodically repeated unit cell comprising a single-unit mesh described by experimentally determined matrices of the substrate lattice vectors, the different unit mesh structures , , and  containing, respectively, one, two, or four TCNQ molecule(s). The Ag(100) surface was modeled as a slab consisting of four atomic layers and separated from its periodic image by a vacuum gap exceeding 90 Å. The bottom two Ag layers were constrained to be fixed; all other atoms were free to move to optimize the structure. The coordinates of the atoms in the bottom two layers of the Ag slab were constrained to the bulk truncated structure of Ag and the positions of the adsorbate and top two layers of the substrate were allowed to relax. In the case of PBE+vdW^surf^, we exclude interactions between Ag atoms and define screened C_6_ coefficients as provided by Ruiz et al.^59^ The Brillouin zone was sampled with an 8 × 8 × 1 Monkhorst–Pack^60^*k*-grid and the geometries were optimized to below a force threshold of 0.025 eV Å^–1^. FHI-aims employs an all-electron numeric atomic orbital basis with tiered default basis set and integration grid specification. All equilibrium structures were optimized with the default “light” basis set, followed by optimization with “tight” basis. In the case of work function calculations, we made a small change to the truncation distance of the Ag atom (the “cut_pot” value) from default 4 to 6 Å. All output files have been deposited as a data set in the NOMAD repository 10.17172/NOMAD/2022.07.28-1 and are freely available.

## 3 Results and Discussion

### 3.1 Experimental Characterization of Co-Adsorption Phases

**Figure 1:**
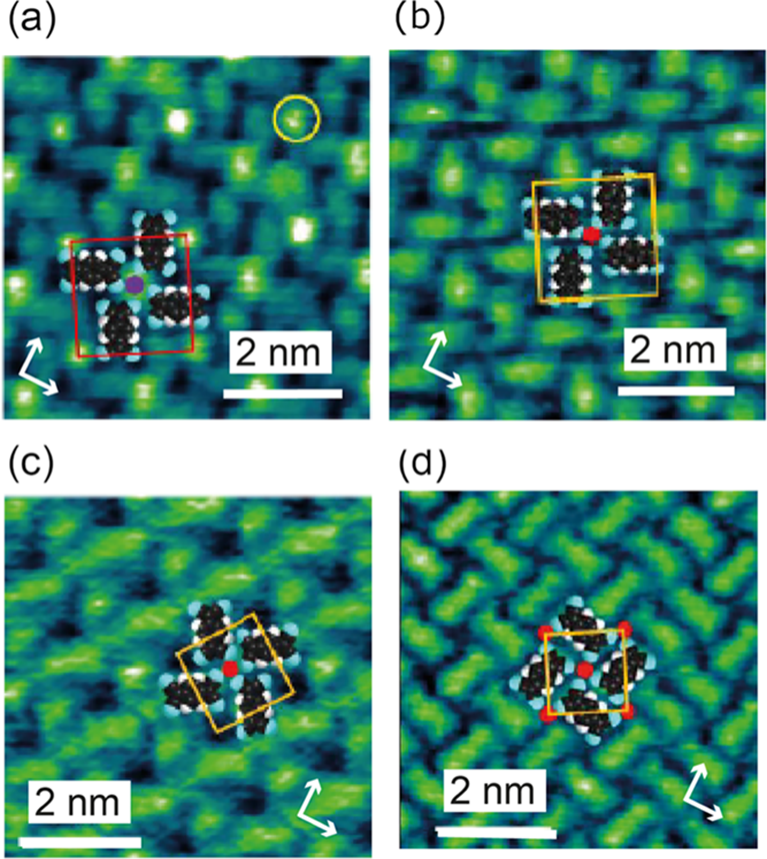
Experimental STM constant tunneling current images of each of the investigated coadsorbed phases. (a)  Cs(TCNQ)_4_, (b)  K(TCNQ)_4_, (c)  K(TCNQ)_2_, (d)  KTCNQ. In each case, the unit mesh and a simple schematic of the implied metal–organic structure are overlaid. Arrows show the <110> surface azimuthal directions, while a bright feature attributed to a Cs atom is circled in (a). Tunneling conditions of sample voltage and tunneling current are (a) −1.3 V, 145 pA, (b) −0.9 V, 250 pA, (c) −1.0 V, 250 pA, and (d) −0.6 V, 300 pA.

As previously reported in an STM investigation by Abdurakhmanova et al.,^50^ coadsorption of Cs and TCNQ on Ag(100) leads to the formation of an ordered phase in which bright atomic-scale features attributed to Cs atoms are each surrounded by four TCNQ molecules arranged like the four vanes of a windmill. This type of “windmill” ordering is seen in other TCNQ adsorption phases involving other coadsorbed metal atoms (e.g., Mn^61^) but also in the absence of co-deposited metal adatoms.^20,62,63^ The ordering of this phase was reported to correspond to the matrix , while the STM images indicate a stoichiometry of Cs(TCNQ)_4_. Our STM and LEED measurements confirm these observations, the constant tunneling current STM image being shown in Figure 1a while the experimental LEED pattern and its simulation are shown in Figure S1. As shown in Figure 1b, a  K/TCNQ coadsorption phase also exists and the STM images indicate identical lateral ordering of the TCNQ molecules to that in the comparable Cs(TCNQ)_4_ phase. It is notable, however, that although the Cs atoms are imaged brightly in the STM (Figure 1a), the K atoms are not imaged in this way. This inability to uniquely identify coadsorbed alkali metal adatoms from STM images has also been seen in alkali/TCNQ layers on Ag(111)^64,65^ but we infer from the identical arrangement of the TCNQ molecules that this phase has the comparable K(TCNQ)_4_ stoichiometry.

Two further KTCNQ coadsorption phases were observed with increasing K coverage leading to indicated stoichiometries of K(TCNQ)_2_ and KTCNQ, with associated matrices of  and , respectively; the corresponding STM images are shown in Figure 1c,d, respectively, while their LEED patterns are shown in Figure S1. Table S1 summarizes the properties of all four detected coadsorption phases. Note that the ordered KTCNQ phase was only observed after annealing to ∼300 °C for a few minutes of a K(TCNQ)_2_ surface onto which additional K had been deposited. It should be noted that the DFT study of Floris et al.^42^ assumed that a  phase, shown experimentally to exist for Cs and TCNQ coadsorption on Ag(100)^50^ also existed for coadsorption of Li, Na, and K with TCNQ. Our results demonstrate that this assumption is valid for K but there appears to be no equivalent phase with coadsorbed Na, although a NaTCNQ  phase equivalent to the KTCNQ phase does exist.^66^ No experiments were performed with Li-TCNQ coadsorption.

**Figure 2:**
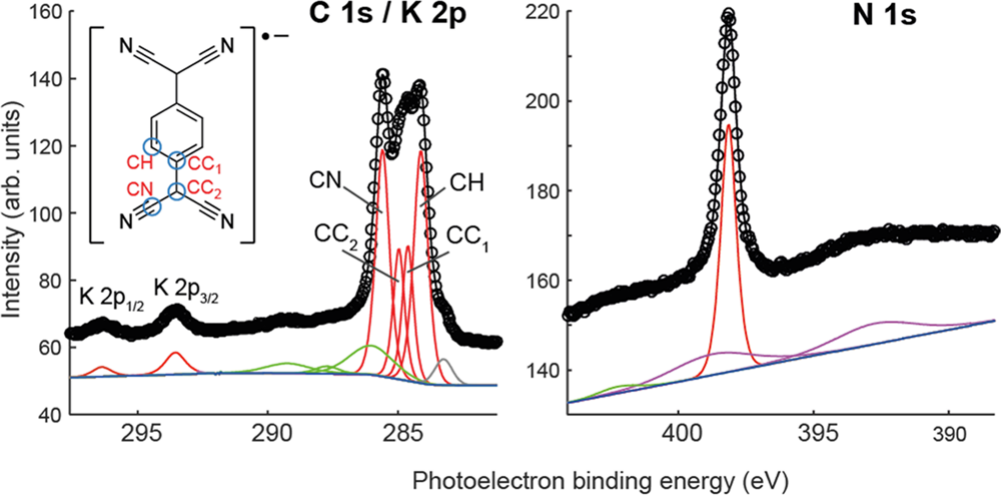
C 1s/K 2p and N 1s SXP spectra recorded from the KTCNQ_4_ phase at photon energies of 435 and 550 eV, respectively. The main photoemission peaks (including the different chemically shifted C 1s peaks) are shown in red. Satellites are shown in green while the loss satellites of the Ag 3d emission are shown in purple.

Additional characterization of these coadsorption phases is provided by the SXP spectra, the spectra from the K(TCNQ)_4_ phase being shown in Figure 2. Similar spectra, recorded from the Cs(TCNQ)_4_ and K(TCNQ)_2_ phases, are presented in Figure S2. No SXPS data were acquired from the KTCNQ phase. The absolute and relative photoelectron binding energies of the chemically distinct C 1s components are characteristic of a negatively charged TCNQ molecule^39,43^ due to electron transfer from the substrate and/or the alkali atoms. The C 1s spectrum can be fitted with four unique features that are assigned to, in increasing binding energy, C atoms bound to H atoms (CH), aromatic C atoms bound only to other C atoms (CC_1_), nonaromatic C atoms bound only to other C atoms (CC_2_) and C atoms bound to N atoms (CN). Only a single N 1s peak was observed, indicating that the molecule remains intact with all N atoms in chemically similar bonding environments. Note that in the N 1s spectra, two broad features are observed in the background; these relate to loss features from the neighboring Ag 3d SXP peaks.

### 3.2 Quantitative Structure Determination by NIXSW and DFT

To complement this largely qualitative characterization of these systems, we have undertaken experimental quantitative structural measurements using the normal-incidence X-ray standing wave technique (NIXSW).^53^ The general XSW technique^67^ exploits the X-ray standing wave, created by the interference of an incident X-ray wave and the resulting Bragg-reflected wave, which shifts through the crystal as one scans across the Bragg condition by varying either the incidence angle or the photon energy. Monitoring the X-ray absorption rate profile of this standing wave in atoms of interest by measuring the element-specific core-level photoemission allows one to determine the location of the absorbing atom relative to the Bragg diffraction planes.^68^ If the Bragg diffraction planes coincide with the surface termination, as in the experiment presented here, the height of the absorbing atoms can be determined from the unique absorption profile that is acquired. Using core-level photoemission to monitor the absorption provides not only elemental specificity but also chemical-state specificity, distinguishing the heights of C atoms in CH, CN, and CC bonds in adsorbed TCNQ as well as determining the height of the Cs and K atoms. We have recently used this approach to investigate the nature of the K_2_TCNQ coadsorption phase on Ag(111), specifically demonstrating that this phase comprises a 2D charge transfer salt.^43^ The present study of phases of different alkali/TCNQ stoichiometry on Ag(100) broadens our insight into the nature of these coadsorption phases.

The measured NIXSW absorption profiles from the *H* Bragg diffraction planes are fitted uniquely by two parameters, the coherent fraction, *f*_H_, and the coherent position, *p*_H_. In the idealized situation of absorber atoms in a single well-defined site with no static or dynamic disorder, *f*_H_ = 1, and the coherent position corresponds to the absorber height above the extended Bragg diffraction planes in units of the Bragg plane spacing, *d*_H_, leading to a height *D* = (*p*_H_ + *n*)·*d*_H_, where *n* is an integer (usually 0 or 1) chosen to ensure interatomic distances are physically reasonable. For Ag, *d*_(200)_ = 2.04 Å. The influence of thermal vibrations of substrate and adsorbate atoms, and possible surface corrugation, can reduce the value of *f* by up to 20–30%, but values of *f* significantly lower than 0.70 must be attributed to two or more distinctly different contributing values of *D*.^69^

Table 1 shows the values of these structural parameters obtained from NIXSW measurements on the K(TCNQ)_4_, K(TCNQ)_2_, and Cs(TCNQ)_4_ phases; no NIXSW measurements were acquired from the KTCNQ phase. Notice that at the higher photon energy of the (200) Bragg reflection (∼3 keV compared to ∼0.4 keV) the spectral resolution of the C 1s photoemission was not sufficient to resolve the distinct CC_1_ and CC_2_ components seen in the SXP spectrum of Figure 2, so these were treated as a single component. All coherent fractions fall within the range consistent with the relevant atoms occupying a single well-defined height above the surface, with the notable exception of the CN and N atoms in the K(TCNQ)_2_ phase. The low *f* value for the N atoms in this case clearly indicates that these N atoms occupy at least two distinctly different heights above the surface, in which case the corresponding *D* value is a weighted mean of the true heights. A height variation of the N atoms must lead to the same qualitative effect, but weaker, for the CN atoms bonded to the N atoms. The heights of all of the molecular components in the KTCNQ_4_ phase are essentially identical to those in Cs(TCNQ)_4_, (and to the values for pure TCNQ adsorption^37^) although in the K(TCNQ)_2_ phase, the central quinoid ring appears to be about 0.1 Å higher.

**Table 1:** Experimental NIXSW (200) Structural Parameter Values, Coherent Fraction *f*, and Coherent Position, Here Converted to a Height Value *D*, for the Three Coadsorption Phases Investigated by This Technique^a^

K(TCNQ)_4_			K(TCNQ)_2_			Cs(TCNQ)_4_		
	*f*	*D* (Å)		*f*	*D* (Å)		*f*	*D* (Å)
CH	0.68(10)	2.72(5)	CH	0.70(10)	2.81(5)	CH	0.63(10)	2.74(5)
CC	0.83(10)	2.64(5)	CC	0.70(10)	2.66(5)	CC	0.80(10)	2.63(5)
CN	0.69(10)	2.53(5)	CN	0.53(10)	2.69(5)	CN	0.71(10)	2.53(5)
N	0.76(10)	2.38(5)	N	0.32(10)	2.57(5)	N	0.78(10)	2.38(5)
K	0.76(10)	3.75(10)	K	0.76(10)	3.61(5)	Cs	0.74(10)	4.08(5)

a Precision estimates (in units of the least significant figure) are shown in parentheses.

Notice, that the experimental precision estimates are random errors arising from the computational fitting and take no account of possible systematic errors. An additional systematic error may be associated with the *D* value for the K atoms in the K(TCNQ)_4_ phase due to the difficulty of separating the weak K 2p emission from the underlying C 1s satellite features. The coverage of K atoms in this adsorption phase is extremely low (0.028 ML, where 1 ML corresponds to one atom or molecule per surface layer Ag atom). Of course, the coverage of Cs atoms in the Cs(TCNQ)_4_ phase has this same low value, and the Cs 3d peaks overlap the (broad) plasmon loss feature of the Ag 3s emission, but extrapolation of tabulated photoionization cross sections^70^ indicates that the value for the Cs 3d state is an order of magnitude larger than that of the K 2p state at the (200) Bragg reflection energy of ∼3 keV; separation of the Cs 3d and the Ag 3s loss peaks is also eased by the fact that the Cs 3d peaks are much narrower.

**Figure 3:**
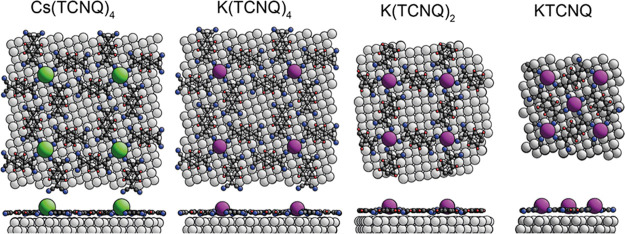
Plan and side view of the four coadsorbed phases as predicted by PBE+vdW^surf^ (Cs/K^+^): Cs(TCNQ)_4_, K(TCNQ)_4_, K(TCNQ)_2_, and KTCNQ on Ag(100). Cs atoms are indicated by the green spheres. K atoms are indicated by purple spheres.

Long-range dispersion-corrected DFT calculations were performed for all four of the coadsorption phases of Figure 1 to establish the optimum structural parameters of the models, which can be compared to our experimental NIXSW measurements. These DFT calculations employed the PBE functional^71^ to evaluate exchange-correlation accompanied by three flavors of dispersion schemes: the standard vdW^surf^ method,^59^ the vdW^surf^ method in which the C_6_ coefficients of Cs and K have been rescaled to account for the change in atomic polarizability upon cation formation (vdW^surf^ (Cs/K^+^)), and the nonlocal many-body dispersion method^49^ (PBE+MBD-NL) as implemented in FHI-aims.^55^ Complete comparisons of the experimental and predicted NIXSW parameters for each adsorption structure can be found in Tables S2–S5 of the Supporting Information (SI). The optimized adsorption structures determined by the DFT calculations for the coadsorption systems on Ag(100) investigated here (shown in Figure 3) involve an alkali atom (Cs or K) coadsorbed with different numbers of TCNQ molecules, according to the experimentally inferred stoichiometries of 1:4 in (Cs(TCNQ)_4_ and K(TCNQ)_4_), 1:2 in (K(TCNQ)_2_) and 1:1 in KTCNQ, respectively. The adsorption motif of the TCNQ molecules is reminiscent of windmill vanes lying on the substrate with the alkali atom at the center, fourfold-coordinated by cyano groups of neighboring TCNQ molecules.

In the case of Cs(TCNQ)_4_ and K(TCNQ)_4_, our findings are in accordance with the structures proposed by Floris and colleagues^42^ for M(TCNQ)_4_ (M = Li,Na,K,Cs). Three of the peripheral cyano groups of each molecule are bent down toward the surface with the cyano group closest to the alkali atom pointing slightly upward toward the alkali atom, leading to N atoms having two different heights above the surface. The same structural feature is found in the K(TCNQ)_2_ phase, but in this phase, there is an equal number of “up” and “down” N atoms, leading to a more significant decrease in the N coherent fraction value. Alkali atoms are fourfold-coordinated to cyano groups in all of the 1:4 and 1:2 stoichiometry phases, the main difference being that TCNQ molecules bridge neighboring alkali atoms in the K(TCNQ)_2_ phase, the alkali atom contacting two cyano groups on opposite ends of the molecule. The KTCNQ phase, in which K and TCNQ are adsorbed in a 1:1 ratio, is more closely packed and rigid relative to other phases as all four cyano groups of each molecule are in direct contact with a K atom.

**Figure 4:**
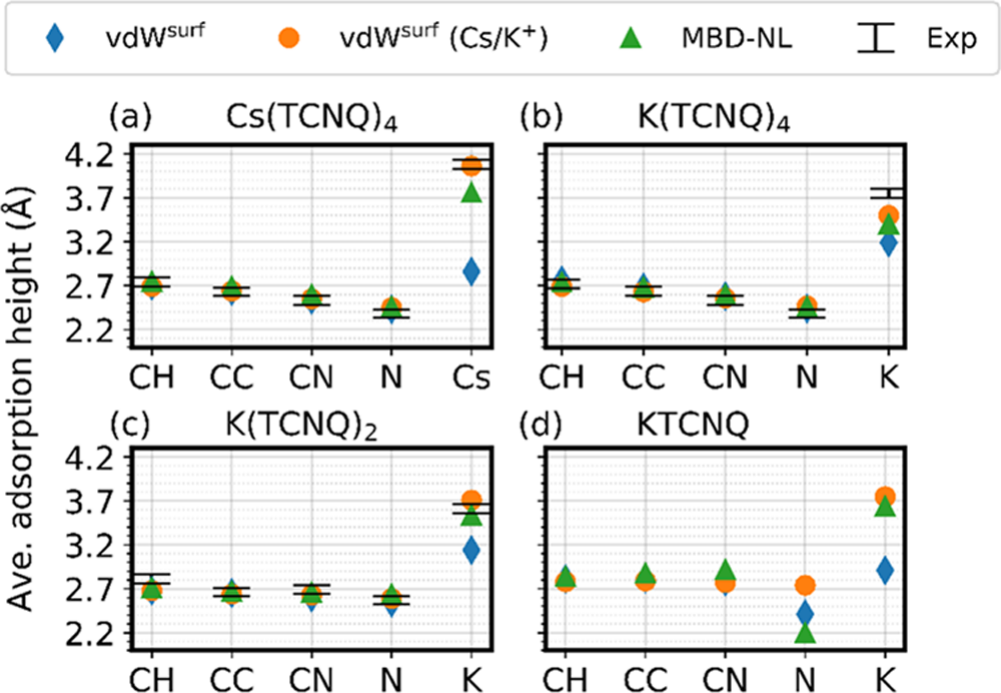
Atomic heights derived from dispersion-inclusive DFT calculations using the three separate dispersion schemes employed in this work. The experimental NIXSW values are shown with black bars indicating the estimated error ranges.

**Figure 5:**
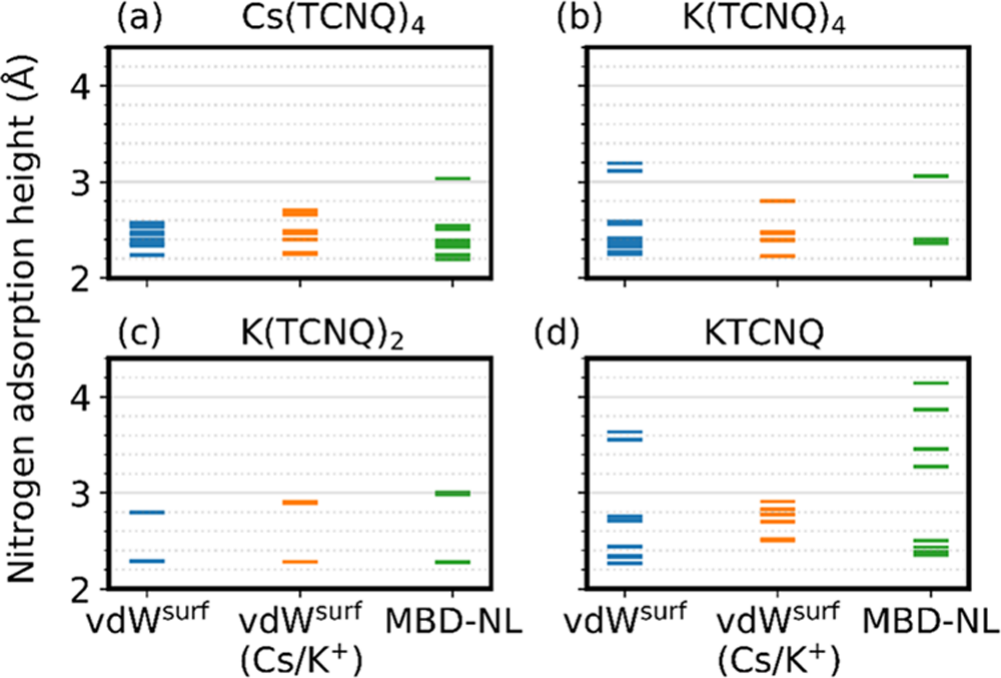
Distribution of nitrogen atom adsorption heights as predicted by different dispersion-corrected DFT methods for (a) Cs(TCNQ)_4_, (b) K(TCNQ)_4_, (c) K(TCNQ)_2_, and (d) KTCNQ. Each bar corresponds to the adsorption height of one nitrogen atom.

Summarized in Table 2 and depicted in Figure 4 is a comparison of the coherent positions (expressed as heights) for the alkali atoms, the N atoms, and the experimentally distinguishable carbon atoms within the TCNQ molecules (defined in Figure 2) determined experimentally by NIXSW and calculated by DFT using different long-range dispersion schemes. Agreement between the experimental and computed *D* values within the experimentally estimated precision is generally good. A comparison of the measured and calculated coherent fractions is shown in Tables S2–S5. Notice that the *f*_theory_ values shown in these tables are determined only by the small variations in height of symmetrically inequivalent atoms in the optimized structures; they take no account of static and dynamic disorder, so the measured values can be expected to be up to ∼30% smaller than the theoretical values due to the effects of static and dynamic disorder perpendicular to the surface.^69^ While most of the experimental and predicted values of the coherent fractions are consistent with a single height, or only a narrow range of heights, of the contributing atoms, in the K(TCNQ)_2_ phase, the significantly lower measured coherent fraction value for the N atoms (0.32 ± 0.10) suggests that there must be significant occupation of at least two different heights of these atoms. If one assumes that the coherent fraction for the N atoms includes a decrease of up to 30% due to static and dynamic disorder, then the maximum expected coherent fraction that could be predicted by the DFT simulations would be 0.46 ± 0.14. With this in mind, the DFT simulations reproduce this expected coherent fraction well (0.58 and 0.45 with vdw^surf^ (K^+^) and MBD-NL, respectively). Indeed, the DFT structural models for this phase do show equal occupation of two contributing heights of the N atoms differing by 0.52–0.72 Å in the different methods of dispersion correction. This height variation arises as N atoms that are adjacent to, and bonding to, the alkali atoms sit higher above the surface than N atoms that are not adjacent to alkali atoms. The range of different N atom heights predicted for the different phases by the two alternative DFT methods is shown in Figure 5. The MBD-NL method always predicts a broader range of N atom heights than the vdW^surf^ method, despite both methods predicting very similar average adsorption heights.

**Table 2:** Comparison of the Experimentally Determined Values of the NIXSW Coherent Position, Converted into Atomic Heights, with the Atomic Heights Relative to the Average Outermost Ag(100) Layer Found in the Results of DFT Calculations for the Three Alkali/TCNQ Adsorption Phases Investigated^a^

	Cs(TCNQ)_4_			K(TCNQ)_4_		
species	*D* (Å) – Exp	*D* (Å) – PBE+vdW^surf^ (Cs/K^+^)	*D* (Å) – PBE+MBD-NL	*D* (Å) – Exp	*D* (Å) – PBE+vdW^surf^ (Cs/K^+^)	*D* (Å) – PBE+MBD-NL
C–H	2.74(5)	2.69	2.75	2.72(5)	2.69	2.74
C–C	2.63(5)	2.64	2.69	2.64(5)	2.63	2.68
C–N	2.53(5)	2.55	2.60	2.53(5)	2.56	2.60
N	2.38(5)	2.45	2.46	2.38(5)	2.47	2.46
K/Cs	4.08(5)	4.06	3.76	3.75(10)	3.50	3.40

	K(TCNQ)_2_			KTCNQ		
C–H	2.81(5)	2.68	2.71	-	2.78	2.84
C–C	2.66(5)	2.64	2.67	-	2.79	2.88
C–N	2.69(5)	2.63	2.66	-	2.77	2.92
N	2.57(5)	2.59	2.64	-	2.74	2.20
K	3.61(5)	3.71	3.54	-	3.75	3.64

a Precision estimates (in units of the least significant figure) are shown in parentheses. All theoretical values reported are calculated at the PBE+vdWsurf (Cs/K^+^) and PBE+MBD-NL levels. No experimental NIXSW data exists for the KTCNQ/Ag(100) phase.

All of the dispersion correction schemes show good agreement with the experimental NISXW layer spacings for the C and N species, as shown in Tables 2 and S2–S5 that present theoretical heights obtained from all three methods. However, the alkali atom adsorption height proved to be much more sensitive to the dispersion scheme employed. Figure 4 displays the height of different atomic species for all of the coadsorption phases investigated here, derived from theoretical calculations employing the three different dispersion schemes together with the experimental values. The values derived from PBE+vdW^surf^ show the worst agreement in alkali atom adsorption height compared to the experimental values. In all cases, the rescaled scheme PBE+vdW^surf^ (Cs/K^+^) shows the best agreement for all of the chemically distinct species. Notably, the Cs height is in good agreement with the experimental value due to our procedure of rescaling the relevant C_6_ dispersion coefficient for the alkali atom; in the absence of rescaling (i.e., for the standard PBE+vdW^surf^ scheme) the Cs height is 1.22 Å lower than experiment. Similarly, the K height in phases K(TCNQ)_4_ and K(TCNQ)_2_ without rescaling are 0.56 and 0.79 Å lower than experiment; these discrepancies are significantly reduced when rescaling is employed (to 0.25 and 0.10 Å, respectively). PBE+MBD-NL outperforms the default version of vdW^surf^, but not the vdW^surf^ (Cs/K^+^) approach. It determines the structural parameters in good agreement with our rescaled and experimental results for the molecular constituent atoms, but also for alkali atom heights. However, an important feature of PBE+MBD-NL is that it includes beyond-pairwise dispersion interactions, which are able to correct for the known overestimation of adsorption energies of PBE+vdW^surf^.^45,72^ In the case of PBE+vdW^surf^ (Cs/K^+^), the manual parameter rescaling accounts for the effect of charge transfer on atomic polarizability. The MBD-NL method supposedly automatically accounts for this effect.

### 3.3 Effect of Overlayer Composition on Stability

In our earlier study of coadsorption of K atoms with TCNQ on the Ag(111) substrate,^43^ we found that the overlayer takes the form of a two-dimensional salt rather than a strongly surface-bound adlayer. This conclusion was reached through a decomposition of the energetic driving forces of adlayer formation and an analysis of the charge distribution. Here, we explore the extent to which this same effect is present on Ag(100) in the four different studied coadsorption phases. Specifically, we ask: what is the effect of changing the coadsorbed alkali atom (Cs vs. K), and what is the effect of changing the alkali/TCNQ ratio? These provide “experimentally tuneable parameters,” so it is important to know how they affect the stability and electronic properties of the interface.

**Figure 6:**
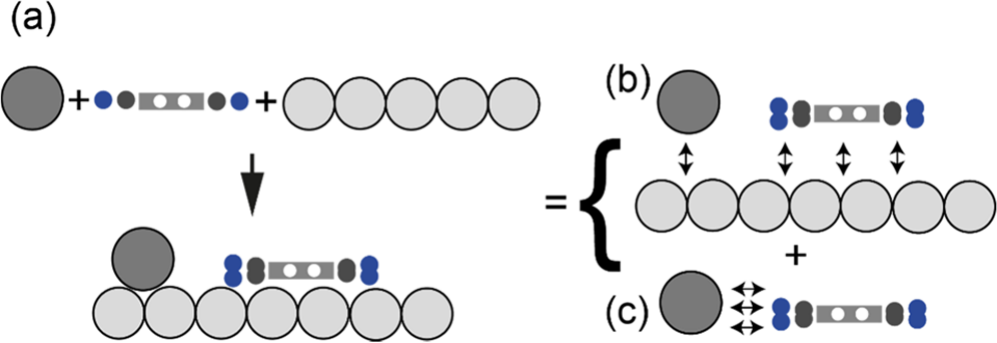
Schematic depiction of the energetic contributions to the adsorption energy of the different adsorbates on Ag(100). (a) Three components of the total coadsorbed system: molecule, alkali atom (dark gray), and metal substrate, before and after coadsorption (below arrow). (b) Schematic representation of the adsorbate overlayer–substrate interaction and (c) intra-adsorbate interaction. Double-headed arrows indicate the interactions that are included in each contribution. The adsorbed molecules are shown with both ends twisted, as is found to be the case in K(TCNQ)_2_.

Figure 6a shows a schematic representation of the components of each coadsorption phase on Ag(100), namely, alkali atoms, molecular adsorbates, and the silver surface. Within the network, we can decompose the total adsorption energy into two contributions: the adsorbate–substrate interaction and the intra-adsorbate interactions, shown in Figure 6b,c, respectively. The total adsorption energy, *E*_ads_, was calculated using eq 1 (below), in which *E*_tot_ is the total energy of the coadsorbed network, *E*_adlayer_ is the total energy of the combined alkali and TCNQ adsorbate layer (frozen in the adsorption geometry as a free-standing layer with the surface removed), *E*_surface_ is the total energy of the optimized bare Ag(100) surface, *E*_Cs,K_ is the total energy of the isolated neutral gas-phase alkali atom, and *E*_TCNQ_ is the total energy of a relaxed gas-phase TCNQ molecule. The first term in square brackets in eq 1 is the strength of adlayer–substrate interaction (as shown in Figure 6b), while the second term in square brackets describes the cohesive energy of the free-standing alkali-molecule layer (shown in Figure 6c). Both terms together yield the adsorption energy associated with bringing all components together.
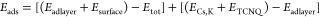
1

The results, summarized in Table 3, show the total adsorption energies per unit surface area of the coadsorbed networks calculated at both the PBE+vdW^surf^ (Cs/K^+^) and PBE+MBD-NL level of theory. While both methods predict the same trends, the MBD-NL adsorption energies are considerably reduced compared to the pairwise scheme. Irrespective of the level of theory, the K(TCNQ)_4_ and Cs(TCNQ)_4_ phases are basically isoenergetic within our numerical tolerance. By increasing the alkali concentration from 1:4 to 1:2 and to 1:1, the adsorption energies increase monotonically, with the 1:1 phase being the most energetically stable.

**Table 3:** Total Adsorption Energies, *E*_ads_, Per Unit Surface Area for Each System Calculated at PBE+vdW^surf^ (Cs/K^+^) and PBE+MBD-NL Levels

	Cs(TCNQ)_4_	K(TCNQ)_4_	K(TCNQ)_2_	KTCNQ
PBE+vdW^surf^ (Cs/K^+^) (eV nm^–2^)	4.95	4.98	5.39	8.57
PBE+MBD-NL (eV nm^–2^)	4.16	4.17	4.60	7.61

**Figure 7:**
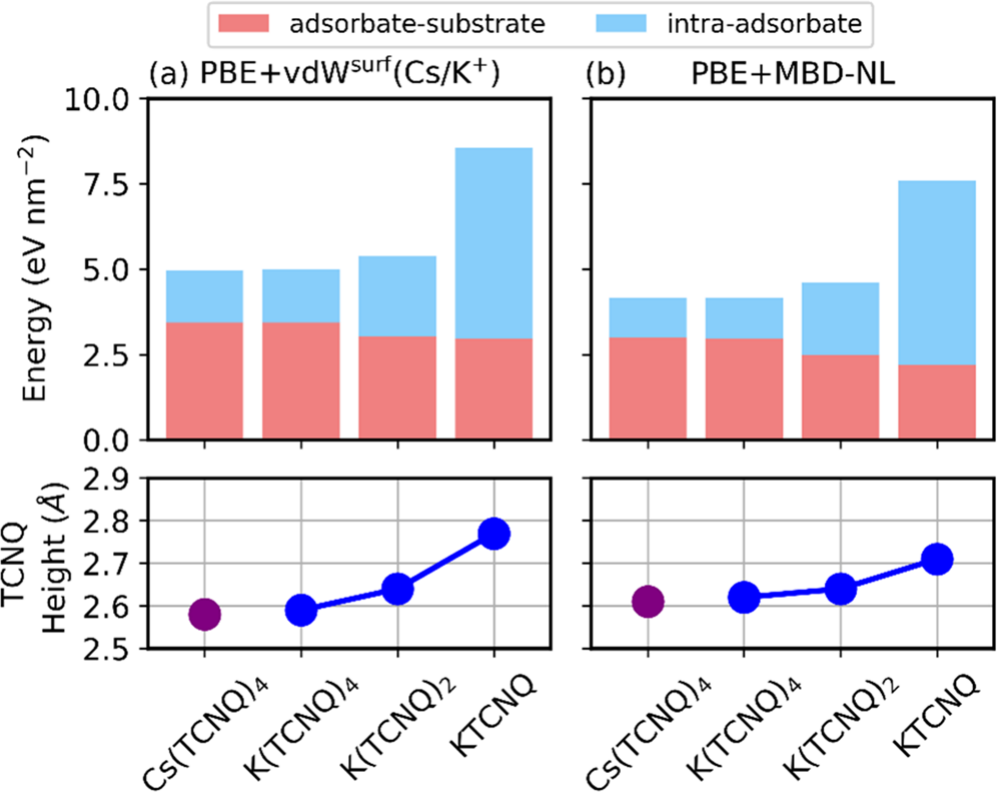
Bar plots showing energetic contributions of (light red) adsorbate–substrate and (light blue) intra-adsorbate interactions as determined by (a) PBE+vdW^surf^ (Cs/K^+^) and (b) PBE+MBD-NL. All values determined by eq 1. (Bottom) Average height of C and N atoms in TCNQ molecules.

Figure 7 shows a histogram of the absolute adsorption energies per unit surface area broken down into the two contributions shown in eq 1. Both 1:4 networks exhibit the same breakdown with the adsorbate–substrate interaction presenting the largest contribution to the overall adsorption energy (around 70%). This is predicted by both PBE+vdW^surf^ (Cs/K^+^) (Figure 7a) and PBE+MBD-NL (Figure 7b) methods. This can be attributed to the fact that Cs(TCNQ)_4_ and K(TCNQ)_4_ show similar optimized adsorption structures (see Figure 2 and Table 1), with similar lateral and perpendicular arrangements at the surface. Both networks adopt a TCNQ windmill vane motif surrounding a central alkali atom. In addition, the alkali atom in both phases occupies the same hollow site with respect to the underlying surface. (Alternative registries were explored in the DFT calculations but were energetically less favorable and failed to account for the discrepancy between the calculated and experimentally determined K atom height in the K(TCNQ)_4_ phase). In the lower panels of Figure 7, the average adsorption height of TCNQ molecules within the networks is shown as a function of the alkali/TCNQ stoichiometry and choice of alkali atom. In both M(TCNQ)_4_ phases and the K(TCNQ)_2_ phase, the cyano groups are twisted. The N atoms close to an alkali atom are higher above the surface than those not adjacent to an alkali atom, which are bent downward toward the underlying Ag(100) surface to maximize interactions with the substrate.

The stability of each network is also dependent upon the constituent stoichiometry of donor and acceptor. When changing the donor–acceptor ratio from 1:4 to 1:2 and to 1:1 for KTCNQ adlayers, the total adsorption energy increases. As shown in Figure 7, this increase can be attributed mainly to the increase of cohesive interactions within the 2D-MOF layer. The increase corresponds to a doubling of this energy contribution from a stoichiometry of 1:4 to 1:2 (1.19–2.49 eV nm^–2^ for MBD-NL) and a further doubling from 1:2 to 1:1 (2.49–5.41 eV nm^–2^). This occurs at the slight expense of the adsorbate–substrate interaction, which is correlated with an increase in average adsorption height of the TCNQ molecule (bottom panel of Figure 7). Although the 2D-MOF cohesive energy accounts for only 29% of the total adsorption energy for the 1:4 alkali/TCNQ ratio, this increases to 54% for 1:2 and to 71% for 1:1. The increasing concentration of alkali atoms leads to a strengthening of the bonding within the 2D-MOF layer. At the same time, the adsorption height of the overlayer above the surface increases, which naturally leads to a slight reduction of the interaction between the 2D-MOF layer and the substrate. The findings are consistent with previous work where the presence of the alkali atoms was found to lead to a structural decoupling of TCNQ with an Ag(111) surface.^43^ The observed stability trends are in qualitative agreement with experimental observations as the KTCNQ phase is created by annealing of other phases, which suggests that it is thermodynamically more stable.

Analysis of the charge distribution via Hirshfeld partitioning provides insight into the cause of the changes in interface stability (summarized in Tables S8–S11 in the SI).^73^ When the adsorbate–substrate interaction dominates, as in the case of K(TCNQ)_4_, we find −0.75*e* charge localized on each TCNQ molecule; the total net charge transferred from the surface to the 2D-MOF in this phase is −2.64*e* per unit mesh, almost entirely accounting for the charge on the molecules. In the case of KTCNQ, in which the 2D-MOF cohesive energy dominates, we only find −0.32*e*/TCNQ and almost no charge transfer from the surface (0.01*e* per unit surface mesh). In K(TCNQ)_2_, in which the energetic contributions are more equal, more charge transfer to the molecules (−0.91*e*/TCNQ) is observed, in part from the substrate (−1.42*e* per unit mesh), but in part from transfer within the 2D-MOF. Thus, in the 1:4 and 1:2 stoichiometry phases, the surface acts as an electron donor leading to stronger adsorbate–surface interactions, but in the 1:1 alkali/TCNQ phase, the net charge transfer between the 2D-MOF and the surface is effectively zero. This leads to a decoupling of the 2D-MOF from the surface, whereas the charge balance between TCNQ and the alkali leads to strong ionic bonding within the 2D-MOF. Note that Hirshfeld partitioning is well known to underestimate the charge population on ions. While it is clear from density difference analysis that alkali atoms have a net charge of +1 *e* and are present within the 2D-MOF as cations, Hirshfeld partitioning only predicts a charge of between +0.3 and +0.5*e*.

### 3.4 Effect of Overlayer Composition on Surface Properties

**Figure 8:**
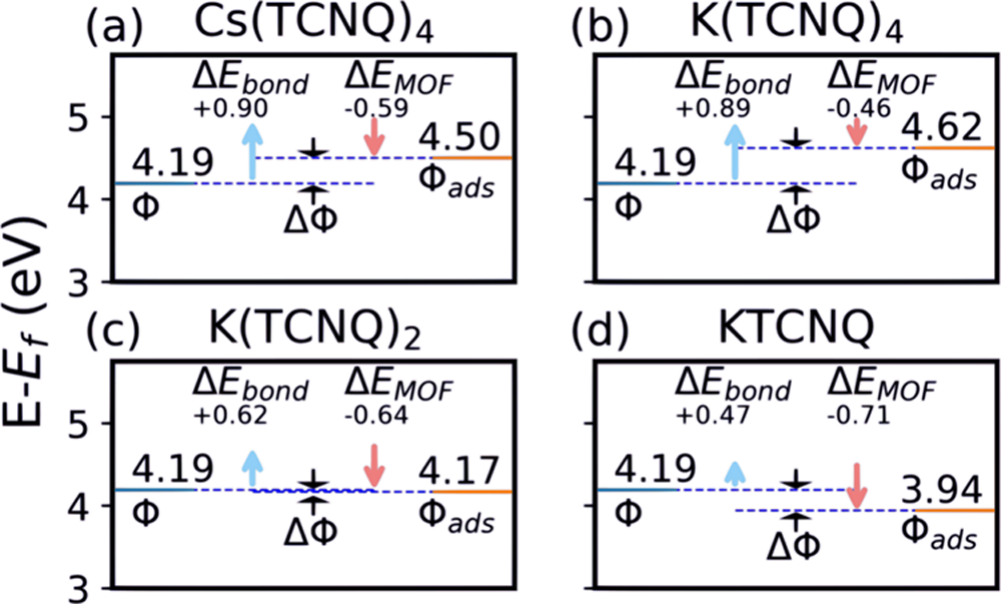
Graphical representation of the results of the theoretical work function analysis for (a) Cs(TCNQ)_4_, (b) K(TCNQ)_4_, (c) K(TCNQ)_2_, and (d) KTCNQ. The work function of the clean surface, ϕ, Δ*E*_bond_ (light blue), Δ*E*_MOF_ (light red), as defined in eq 2, the work function after adsorption, ϕ_ads_ and the change in work function ΔΦ. All values are calculated at the PBE+MBD-NL level.

Adsorbates that are strong electron acceptors (donors) are well known to increase (decrease) the work function at the interface due to charge transfer.^74−76^ The overall change in work function Δϕ due to the adsorption of a molecular overlayer can be decomposed into two terms
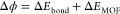
2This includes a contribution due to the dipole density of the 2D-MOF overlayer, Δ*E*_MOF_, and a contribution due to the charge transfer and hybridization of the adsorbate overlayer with the substrate, Δ*E*_bond_. We calculate Δ*E*_MOF_ as the potential drop through the free-standing 2D-MOF and evaluate Δ*E*_bond_ as the difference between Δϕ and Δ*E*_MOF_. We refer to the second term as Δ*E*_MOF_ as it arises from the dipole moment perpendicular to the surface of the complete 2D-MOF rather than just the TCNQ molecules. Figure 8 shows the resulting changes in work function, together with these two separate contributions, relative to that of the clean Ag(100) surface as predicted by PBE+MBD-NL (4.19 eV).

For the 1:4 stoichiometry phases, with four electron-accepting TCNQ molecules and only one electron-donating alkali atom, our PBE+MBD-NL calculations show, as might be expected, that relative to the clean Ag(100) surface there are net increases in the work function (+0.31 and +0.43 eV for Cs(TCNQ)_4_ and K(TCNQ)_4_, respectively). However, increasing the fraction of alkali atoms to obtain the KTCNQ_2_ phase leads to a small decrease in the work function (-0.02 eV), while in KTCNQ there is a significant work function decrease (to -0.25 eV). (see also Figure S4 for PBE+vdW^surf^ (Cs/K^+^) results and Tables S6 and S7 in the SI for tabulated values). By going from a high TCNQ concentration to a 1:1 donor/acceptor ratio, the initial increase in work function upon adsorption ultimately changes to a decrease as dipole effects overwhelm chemical bonding effects.

Δ*E*_bond_ is essentially equal for K(TCNQ)_4_ and Cs(TCNQ)_4_ because the molecular heights are similar in these two structures, as is the charge transfer predicted by Hirshfeld analysis to the TCNQ (Cs(TCNQ)_4_; −0.80*e*/TCNQ, K(TCNQ)_4_; −0.75*e*/TCNQ, see Tables S8 and S9). The main difference in Δϕ for these two phases is in the contribution due to the potential drop Δ*E*_MOF_, as Cs(TCNQ)_4_ has a larger dipole moment than K(TCNQ)_4_. This can be attributed to the fact that Cs is more spatially separated from the molecules in the layer than is K.

## 4 Conclusions

We report the formation and characterization of several two-dimensional metal–organic frameworks composed of electron-donating alkali atoms and the strong electron acceptor molecule, TCNQ, at various donor/acceptor ratios on Ag(100). We determine the adsorption structures via the quantitative experimental technique of NIXSW, complemented by state-of-the-art dispersion-inclusive density functional calculations of the experimentally identified adsorption phases with compositions Cs(TCNQ)_4_, K(TCNQ)_4_, K(TCNQ)_2_ and KTCNQ. In all cases for which NIXSW data was available, we present structural models predicted by DFT that are in good agreement with experimental measurements. Although PBE+vdW^surf^ with rescaled C_6_ coefficients to account for the cationic nature of alkali atoms, vdW^surf^ (Cs/K^+^), provides the most accurate adsorption height predictions, we find that the MBD-NL dispersion correction is not far off from vdW^surf^ (Cs/K^+^) without the need for manual intervention. This shows that PBE+MBD-NL is able to provide robust structure predictions even in the case of strongly charge-separated systems with the additional benefit of mitigating the well-known overbinding of vdW^surf^.

Having arrived at accurate structural models, we used DFT calculations to assess the stability of each phase. The Cs(TCNQ)_4_ and K(TCNQ)_4_ phases show similar energetic stability. In general, the nature and size of the alkali atom do not affect the cohesion of the two-dimensional metal–organic framework that is formed, although Cs, due to its larger size, is found at an elevated height above the plane of the molecular overlayer. By comparing K/TCNQ phases with different donor/acceptor ratios, we find trends in the overall stability of the phases as well as the energetic contributions that arise from adlayer–substrate interaction and from the cohesion within the 2D-MOF. The overall stability of the network increases as the donor concentration is increased, mainly due to an increase in the strength of the ionic cohesive stabilization within the layer. With a 1:1 alkali/TCNQ stoichiometry, the adlayer height increases and the 2D-MOF becomes more decoupled from the substrate than in the 1:4 case. Formation of the 1:4 phases (K(TCNQ)_4_ and Cs(TCNQ)_4_) leads to a strong increase in work function relative to the clean Ag(100) surface, 1:2 leaves the work function virtually unaffected, whereas the 1:1 phase reduces the work function of the Ag(100) surface.

In the context of device design, the results of this study can point to potential routes to exert control over observable properties at the interface via controlled sequential adsorption of alkali atoms and strong acceptor molecules to achieve varying donor/acceptor stoichiometries at the surface. The variation in structural composition has effects on the interface stability and bonding that are mediated by two-dimensional salt formation within the 2D-MOF layer. As a function of donor/acceptor ratio, surface electronic properties are strongly modified as dipole formation and molecular charge transfer vary due to competing interactions between alkali atoms, the substrate, and the acceptor molecule. At lower ratios (1:4) we find characteristic surface-bound adlayers and at the donor–acceptor parity (1:1), we find an organic charge transfer salt layer that is more weakly bonded to the surface. In the latter case, the surface dipole is mostly governed by the potential drop across this salt layer. As alkali atom deposition is an experimentally viable technique in thin-film manufacturing, our results show the significant structural and electronic changes that can be affected by controlled alkali atom doping.

## Supporting Information Available

Additional detailed experimental results, experimental heights, additional height data from calculations, theoretical work function and charge redistribution data, and molecular orbital density of states data (PDF)
