# Supplementary material for: Donor–Acceptor Co-Adsorption Ratio Controls the Structure and Electronic Properties of Two-Dimensional Alkali–Organic Networks on Ag(100)
Source: J Phys Chem C Nanomater Interfaces. 2023 Jan 26;127(5):2716–27. doi: 10.1021/acs.jpcc.2c08688 (PMC9923740; doi:10.1021/acs.jpcc.2c08688)
Supplement: Supplementary file 1 — jp2c08688_si_001.pdf [file jp2c08688_si_001.pdf]

Supporting Information to

Donor-Acceptor Co-Adsorption Ratio Controls Structure and Electronic Properties of Two-Dimensional Alkali-Organic Networks on Ag(100)

B. Sohail[1], P.J. Blowey[2,3], L.A. Rochford[4], P.T.P. Ryan[3,5], D.A. Duncan[3], T.-L. Lee[3], P. Starrs [3,6], G. Costantini[2,4], D.P. Woodruff[2*], R.J. Maurer[1,2*]

(1) Department of Chemistry, University of Warwick, Coventry CV4 7AL, UK

(2) Department of Physics, University of Warwick, Coventry CV4 7AL, UK

(3) Diamond Light Source, Harwell Science and Innovation Campus, Didcot, OX11 0DE, UK

(4) School of Chemistry, University of Birmingham, Birmingham B15 2TT, UK

(5) Department of Materials, Imperial College, London SW7 2AZ, UK

(6) School of Chemistry, University of St. Andrews, St. Andrews, KY16 9AJ, UK

Contents

1. Additional detailed experimental results: LEED, SXPS and NIXSW
2. Experimental heights
3. Additional height data from varying dispersion scheme
4. Lateral registry comparison of K/Cs$(TCNQ)_4$ phases
5. Theoretical Work function data
6. Charge re-distribution data
7. Molecular orbital density of states (MODOS)

* Email: r.maurer@warwick.ac.uk, d.p.woodruff@warwick.ac.uk

1. Additional detailed experimental results: LEED, SXPS and NIXSW

LEED patterns from the 4 different alkali-TCNQ adsorption phases are shown in Figure S1.

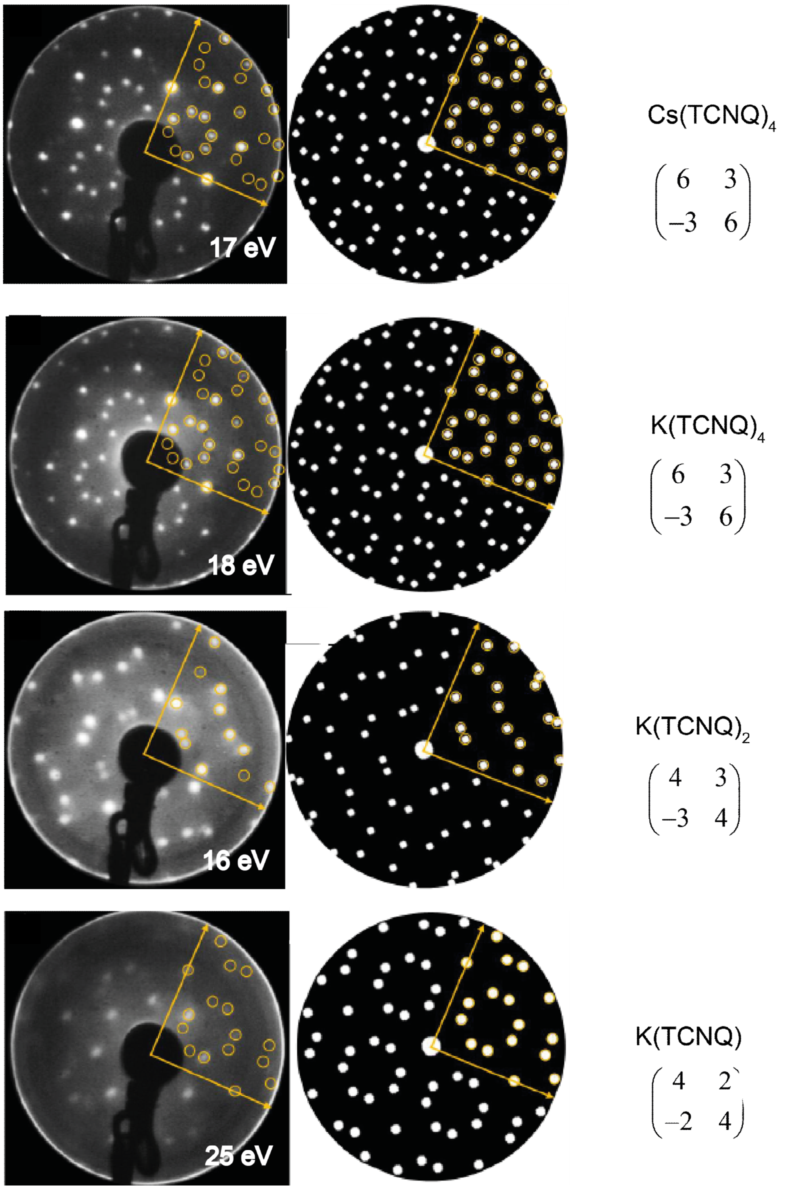

Figure S1. Experimental LEED patterns (left) and simulations of these patterns using the LEEDpat[1] program (right) for each of the 4 different alkali-TCNQ coadsorption phases. Circled in yellow are all the predicted beams in the upper-right hand quadrant, these circles also being repeated in the experimental LEED patterns. The yellow arrows show the <110> directions of the Ag(100) substrate.

A summary of the different phases found is given in Table S1

Table S1 Summary of the different ordered co-adsorption phases of TCNQ and K/Cs found on Ag(100).

Phase descriptor	Matrix	Unit mesh area (Å²)	No of molecules per unit mesh	No of K/Cs atoms per unit mesh	Area per molecule (Å²)	Preparation
$KTCNQ_4$ and $CsTCNQ_4$	$\begin{pmatrix} 6 & 3 \\ -3 & 6 \end{pmatrix}$	375	4	1	94	Alkali deposition onto Ag(100)$\begin{pmatrix} 1 & 4 \\ -3 & -1 \end{pmatrix}$-TCNQ surface
$KTCNQ_2$	$\begin{pmatrix} 4 & 3 \\ -3 & 4 \end{pmatrix}$	210	2	1	105	Additional K deposition onto $KTCNQ_4$ phase
KTCNQ	$\begin{pmatrix} 4 & 2 \\ -2 & 4 \end{pmatrix}$	166	2	2	83	Additional K deposition onto $KTCNQ_2$ and annealing to ~300°C

SXPS data from the $K(TCNQ)_4$ phase are shown in Figure 2 of the main manuscript, while SXPS from the $K(TCNQ)_2$ and $Cs(TCNQ)_4$ phases are shown in Figure S2

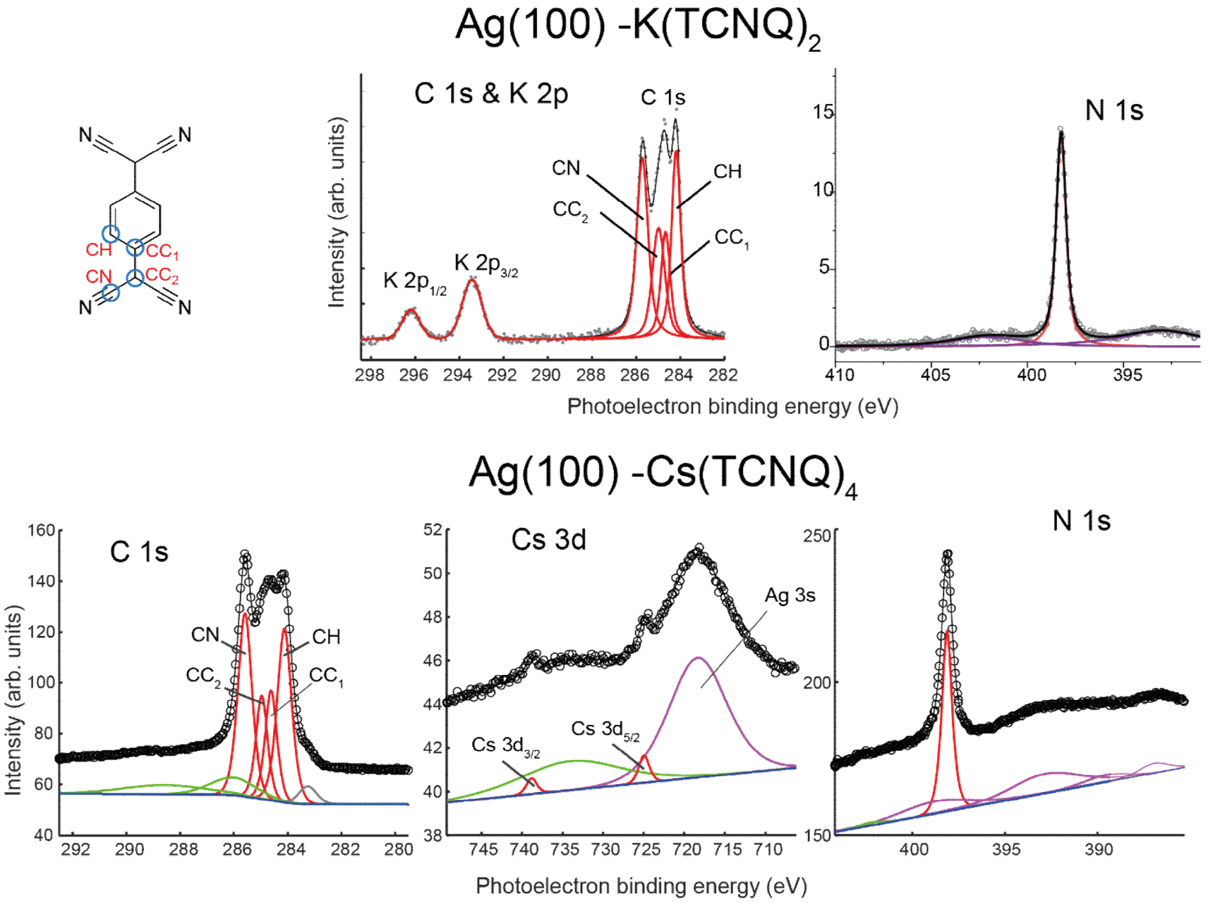

Figure S2 C 1s and K 2p, Cs 3d, and N 1s SXP spectra recorded from the $K(TCNQ)_2$ and $Cs(TCNQ)_4$ phases on Ag(100) at photon energies of 435 eV, 900 eV and 550 eV respectively. The main photoemission peaks (including the different chemically-shifted C 1s peaks) are shown in red. Satellites are shown in green while the plasmon satellites of the Ag 3d emission in the N 1s spectrum are shown in purple. A schematic of the TCNQ molecule shows the labelling of the inequivalent C atoms that are distinguished in the C 1s spectra.

Figure S3 shows a comparison of the raw NIXSW photoemission intensity scans from the three alakli/TCNQ coadsorption phases investigated by this technique with the best-fit theoretical curves, thecorresponding values of the two fitting parameters (coherent fraction and coherent position) being reported in Table 1 of the main paper. The analysis included corrections for the backward/forward asymmetry of the photoemission angular dependence.

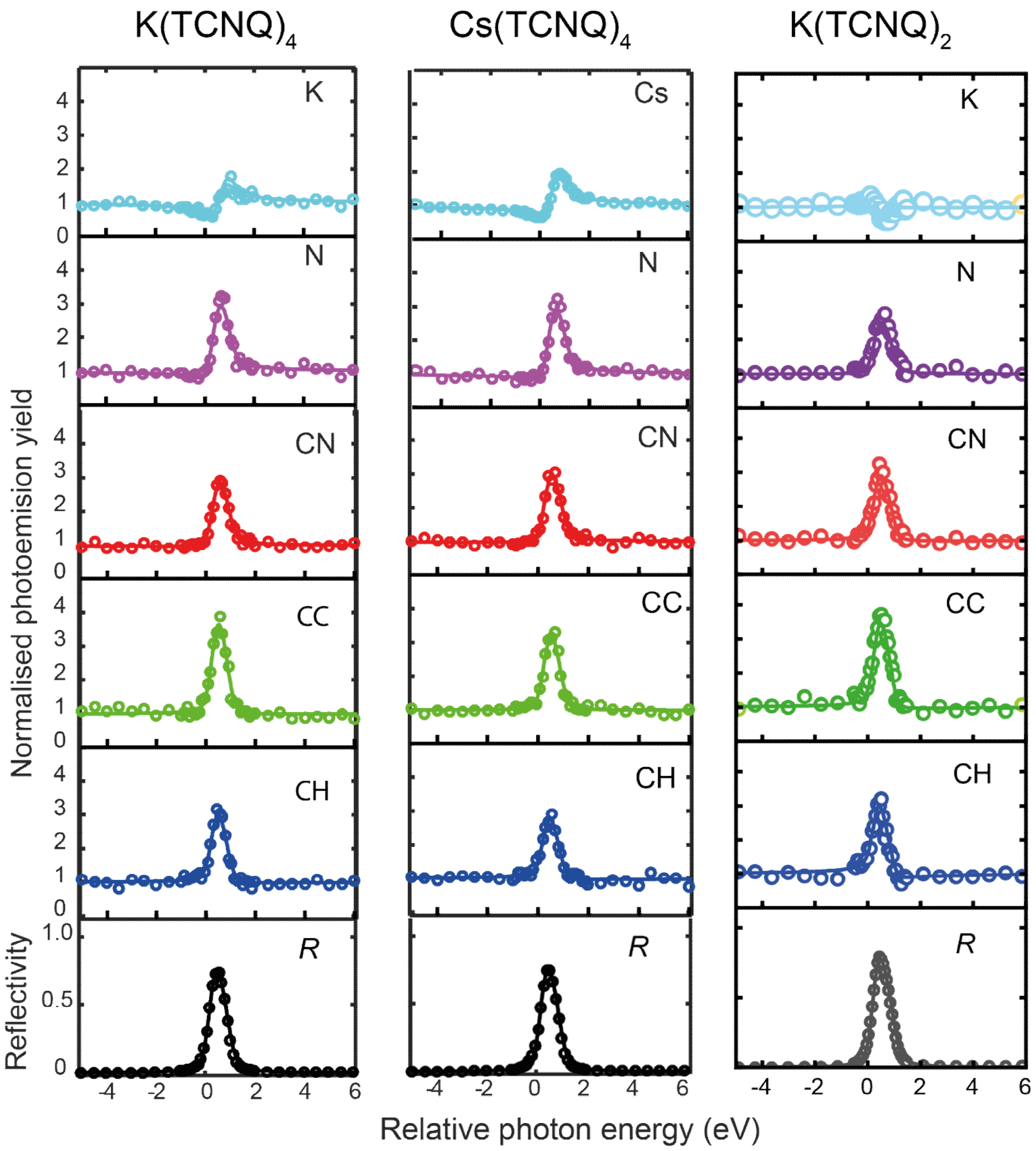

Figure S3 Chemical-state specific NIXSW photoemission data for the (200) Bragg reflection from the $K(TCNQ)_4$, $Cs(TCNQ)_4$ and $K(TCNQ)_2$ surface phases. Experimental data points are shown as circles while the continuous lines are theoretical fits corresponding to the coherent fraction and position values reported in Table 1.

2. Comparison of predicted NIXSW structural parameters using different dispersion schemes

Tables S2-S5 provide a comparison of the experimental NIXSW structural parameters and the predicted values of these parameters determined from DFT calculations for 3 dispersion schemes employed, namely PBE+vdW^{surf}(Cs/K+),[2] PBE+vdW^{surf},[3] and PBE+MBD-NL[4]. Notice that the theoretical values of the coherent fractions, *f*, however, take no account of the static or dynamic disorder, which may be present in the experimental values.

Table S2 Comparison of the experiential NIXSW structural parameters for the Ag(100) Cs(TCNQ)$_4$ phase with the predictions of the three different dispersion schemes.

	f **expt**	***D*** **(Å) expt**	***f*** **vdW^{surf}-Cs$^+$**	***D*** **(Å) vdW^{surf}-Cs$^+$**	***f*** **vdW^{surf}**	***D*** **(Å) vdW^{surf}**	***f*** **MBD-NL**	***D*** **(Å) MBD-NL**
C-H	0.63(10)	2.72(5)	0.99	2.69	0.99	2.70	1.00	2.75
C-C	0.80(10)	2.64(5)	0.99	2.64	0.99	2.64	0.98	2.69
C-N	0.71(10)	2.53(5)	0.97	2.55	0.98	2.54	0.90	2.60
N	0.78(10)	2.38(5)	0.90	2.45	0.93	2.43	0.71	2.46
Cs	0.76(10)	4.08(5)	1.00	4.06	1.00	2.86	1.00	3.76

Table S3 Comparison of the experiential NIXSW structural parameters for the Ag(100) K(TCNQ)$_4$ phase with the predictions of the three different dispersion schemes.

	f **expt**	***D*** **(Å) expt**	***f*** **vdW^{surf}-K$^+$**	***D*** **(Å) vdW^{surf}-K$^+$**	***f*** **vdW^{surf}**	***D*** **(Å) vdW^{surf}**	***f*** **MBD-NL**	***D*** **(Å) MBD-NL**
C-H	0.68(10)	2.72(5)	0.99	2.69	0.99	2.76	1.00	2.74
C-C	0.83(10)	2.64(5)	0.99	2.64	0.98	2.68	0.98	2.68
C-N	0.69(10)	2.53(5)	0.94	2.56	0.90	2.58	0.87	2.60

N	0.76(10)	2.38(5)	0.81	2.47	0.74	2.44	0.66	2.46
K	0.76(10)	3.75(10)	1.00	3.50	0.94	3.19	1.00	3.40

Table S4 Comparison of the experiential NIXSW structural parameters for the Ag(100) $K(TCNQ)_2$ phase with the predictions of the three different dispersion schemes.

	f **expt**	***D*** **(Å) expt**	***f*** **vdW^{surf}-K^+**	***D*** **(Å) vdW^{surf}-K^+**	***f*** **vdW^{surf}**	***D*** **(Å) vdW^{surf}**	***f*** **MBD-NL**	***D*** **(Å) MBD-NL**
C-H	0.70(10)	2.81(5)	1.00	2.68	1.00	2.68	1.00	2.71
C-C	0.70(10)	2.66(5)	1.00	2.64	0.99	2.65	0.99	2.67
C-N	0.53(10)	2.69(5)	0.87	2.63	0.91	2.60	0.83	2.66
N	0.32(10)	2.57(5)	0.58	2.59	0.71	2.54	0.45	2.64
K	0.76(10)	3.61(5)	1.00	3.71	1.00	3.14	1.00	3.54

Table S5 Comparison of the predicted NIXSW structural parameters for the Ag(100) KTCNQ phase obtained using the three different dispersion schemes.

	f **expt**	***D*** **(Å) expt**	***f*** **vdW^{surf}-K^+**	***D*** **(Å) vdW^{surf}-K^+**	***f*** **vdW^{surf}**	***D*** **(Å) vdW^{surf}**	***f*** **MBD-NL**	***D*** **(Å) MBD-NL**
C-H	-	-	1.00	2.78	0.98	2.81	0.92	2.84
C-C	-	-	1.00	2.79	1.00	2.82	0.96	2.88
C-N	-	-	0.98	2.77	0.70	2.78	0.52	2.92
N	-	-	0.91	2.74	0.40	2.41	0.26	2.20
K	-	-	1.00	3.75	0.94	2.91	0.96	3.64

3. Theoretical Work function data

Table S6 Work functions computed for both adsorbate systems and the clean surface. Reported are the work function ϕ, the respective change in work function when compared to the clean substrate $\Delta\phi$, the electrostatic contribution of the work function change from the adsorbate overlayer ΔE_{mol}, and the contribution of the work function change due to the chemical interaction of the overlayer with the metal ΔE_{bond}. The two contributions sum up to the total work function change: $\Delta\phi = \Delta E_{mol} + \Delta E_{bond}$. ΔE_h and

ΔE_e represent the hole and electron injection barriers, respectively. All values calculated at the PBE+vdW^{surf}(Cs/K^+)[2] level.

	**Work function ϕ / eV**	**$\Delta\phi$ / eV**	**ΔE_{mol} / eV**	**ΔE_{bond} / eV**	**ΔE_h / eV**	**ΔE_e / eV**
Ag(100)	4.19					
$Cs(TCNQ)_4$	4.34	0.15	-0.68	0.84	0.63	2.13
$K(TCNQ)_4$	4.52	0.33	-0.50	0.83	0.67	2.10
$K(TCNQ)_2$	4.14	-0.05	-0.65	0.60	0.72	2.00
KTCNQ	3.28	-0.91	-1.27	0.36	0.80	1.86

Table S7 Work functions computed for both adsorbate systems and the clean surface. Reported are the work function ϕ, the respective change in work function when compared to the clean substrate $\Delta\phi$, the electrostatic contribution of the work function change from the adsorbate overlayer ΔE_{mol}, and the contribution of the work function change due to the chemical interaction of the overlayer with the metal ΔE_{bond}. The two contributions sum up to the total work function change: $\Delta\phi = \Delta E_{mol} + \Delta E_{bond}$. ΔE_h and ΔE_e represent the hole and electron injection barriers, respectively. All values calculated at the PBE+MBD-NL[4] level.

	**Work function ϕ / eV**	**$\Delta\phi$ / eV**	**ΔE_{mol} / eV**	**ΔE_{bond} / eV**	**ΔE_h / eV**	**ΔE_e / eV**
Ag(100)	4.19					
$Cs(TCNQ)_4$	4.50	0.31	-0.59	0.90	-	-
$K(TCNQ)_4$	4.62	0.43	-0.46	0.89	-	-
$K(TCNQ)_2$	4.17	-0.02	-0.64	0.62	-	-
KTCNQ	3.94	-0.25	-0.71	0.47	-	-

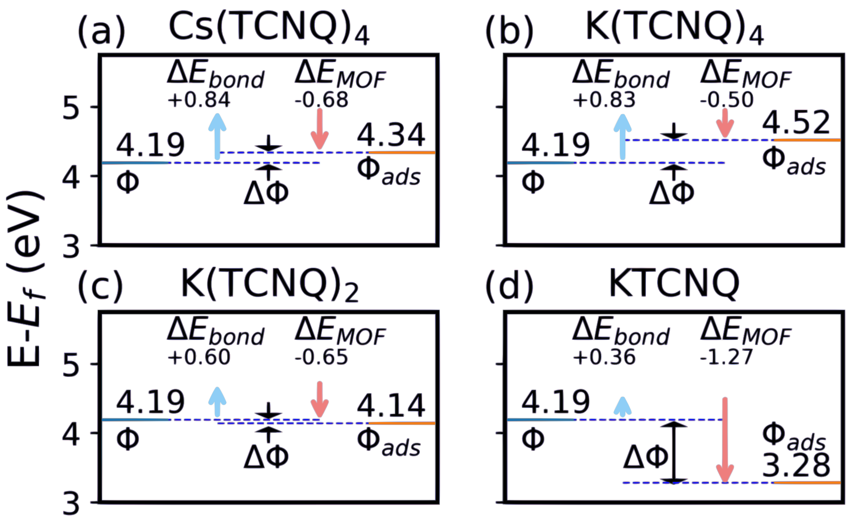

Figure S4- Schematic representation of the theoretical work function analysis. The work function of the clean surface, ϕ, ΔE_{bond}(light blue), ΔE_{mol} (light red), the work function after adsorption, ϕ_{ads} and the change in work function $\Delta\Phi$. All values calculated at the PBE+vdW^{surf}(Cs/K^{+}) level

4. Charge re-distribution data

Table S8 - Hirshfeld charge[5] values for all species in the $Cs(TCNQ)_4$ unit cell. Units in elementary electronic charge *e*

**$Cs(TCNQ)_4$**	**Cs (per atom)**	**TCNQ (per molecule)**	**Substrate (per unit cell)**
Hirshfeld charge	0.51	-0.80	-2.68

Table S9 - Hirshfeld charge[5] values for all species in the $K(TCNQ)_4$ unit cell. Units in elementary electronic charge *e*

**$K(TCNQ)_4$**	**K (per atom)**	**TCNQ (per molecule)**	**Substrate (per unit cell)**
Hirshfeld charge	0.36	-0.75	-2.64

Table S10 - Hirshfeld charge[5] values for all species in the $K(TCNQ)_2$ unit cell. Units in elementary electronic charge *e*

**$K(TCNQ)_2$**	**K (per atom)**	**TCNQ (per molecule)**	**Substrate (per unit cell)**
Hirshfeld charge	0.40	-0.91	-1.42

Table S11 - Hirshfeld charge[5] values for all species in the KTCNQ unit cell. Units in elementary electronic charge e

**KTCNQ**	**K (per atom)**	**TCNQ (per molecule)**	**Substrate (per unit cell)**
Hirshfeld charge	0.31	-0.32	-0.01

5. Molecular orbital density of states (MODOS)

Shown in Figure S5 are the MODOS for all systems calculated with PBE+vdW^{surf}(Cs/K^+). In the case of Cs(TCNQ)$_4$, the former LUMO+1, exhibits broader peaks than K(TCNQ)$_4$ which indicates a more complex hybridisation between the Ag and TCNQ states. All occupied states are very similar in both energetic position and shape. Furthermore, the larger absolute height of Cs compared to K can be partly attributed to the difference in van der Waals radii of each species but also to the increased dipole with increasing van der Waals radii. In the cases of K(TCNQ)$_2$ and KTCNQ, we find very little difference between the MODOS although a slight reduction in (former) HOMO and LUMO energetic positions.

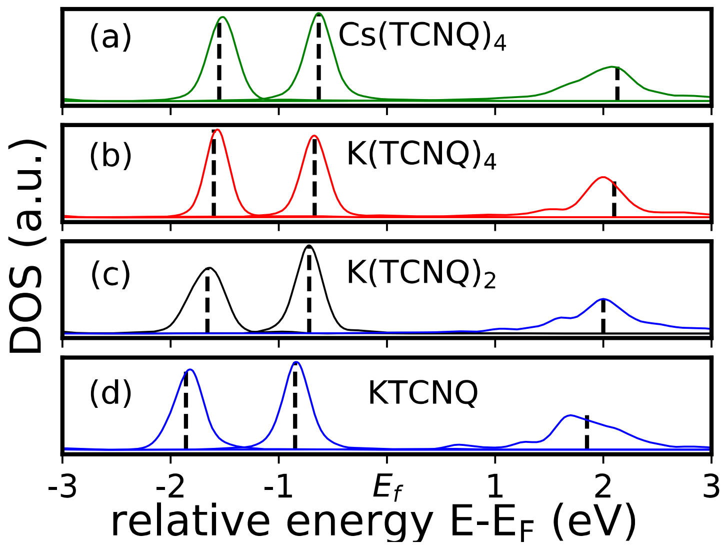

Figure S5 - DOS plots of frontier states of TCNQ in the co-adsorbed system on Ag(100): (a) CsTCNQ$_4$, (b) KTCNQ$_4$, (c) KTCNQ$_2$, (d) KTCNQ (Left to right) filled peaks represent HOMO, and empty peaks represent the (former) LUMO and LUMO+1. Dashed black lines represent the energetic peak position as determined by equation 4 as reported in the main manuscript.

References:

(1) Hermann, K.; Van Hove, M. LEEDpat, 2014.

(2) Blowey, P. J.; Sohail, B.; Rochford, L. A.; Lafosse, T.; Duncan, D. A.; Ryan, P. T. P.; Warr, D. A.; Lee, T.-L.; Costantini, G.; Maurer, R. J.; Woodruff, D. P. Alkali Doping Leads to Charge-Transfer Salt Formation in a Two-Dimensional Metal–Organic Framework. *ACS Nano* **2020**, *14* (6), 7475–7483. https://doi.org/10.1021/acsnano.0c03133.

(3) Ruiz, V. G.; Liu, W.; Zojer, E.; Scheffler, M.; Tkatchenko, A. Density-Functional Theory with Screened van Der Waals Interactions for the Modeling of Hybrid Inorganic-Organic Systems. *Phys. Rev. Lett.* **2012**, *108* (14), 146103. https://doi.org/10.1103/PhysRevLett.108.146103.

(4) Hermann, J.; Tkatchenko, A. Density Functional Model for van Der Waals Interactions: Unifying Many-Body Atomic Approaches with Nonlocal Functionals. *Phys. Rev. Lett.* **2020**, *124* (14), 146401. https://doi.org/10.1103/PhysRevLett.124.146401.

(5) Hirshfeld, F. L. Bonded-Atom Fragments for Describing Molecular Charge Densities. *Theoret. Chim. Acta* **1977**, *44* (2), 129–138. https://doi.org/10.1007/BF00549096.
